# Multimodal, Idiographic Ambulatory Sensing Will Transform our Understanding of Emotion

**DOI:** 10.1007/s42761-023-00206-0

**Published:** 2023-08-10

**Authors:** Katie Hoemann, Jolie B. Wormwood, Lisa Feldman Barrett, Karen S. Quigley

**Affiliations:** 1 Department of Psychology, KU Leuven, Tiensestraat 102, Box 3727, 3000 Leuven, BE Belgium; 2 Department of Psychology, University of New Hampshire, Durham, NH USA; 3 Department of Psychology, Northeastern University, Boston, MA USA; 4 Department of Psychiatry, Massachusetts General Hospital and Harvard Medical School, Cambridge, MA USA; 5 Athinoula A. Martinos Center for Biomedical Imaging, Massachusetts General Hospital, Charlestown, MA USA

**Keywords:** Mobile sensing, Experience sampling, Ecological momentary assessment, Ambulatory peripheral physiology

## Abstract

Emotions are inherently complex – situated inside the brain while being influenced by conditions inside the body and outside in the world – resulting in substantial variation in experience. Most studies, however, are not designed to sufficiently sample this variation. In this paper, we discuss what could be discovered if emotion were systematically studied within persons ‘in the wild’, using biologically-triggered experience sampling: a multimodal and deeply idiographic approach to ambulatory sensing that links body and mind across contexts and over time. We outline the rationale for this approach, discuss challenges to its implementation and widespread adoption, and set out opportunities for innovation afforded by emerging technologies. Implementing these innovations will enrich method and theory at the frontier of affective science, propelling the contextually situated study of emotion into the future.

Emotions are situated and multimodal: they unfold over time as the brain continuously converses with the body and the external world. Given this complexity, instances of the same emotion category (e.g., ‘anger’) are highly variable across individuals as well as within individuals across contexts. This variation is documented by a growing number of studies and meta-analyses of brain activity (e.g., Doyle et al., 2022; Westlin et al., 2023), peripheral physiological activity (e.g., Hoemann et al., 2020; Siegel et al., 2018), facial muscle movements (e.g., Barrett et al., 2019; Durán & Fernández-Dols, 2021) and other behaviors (e.g., Tsai et al., 2006; Wake et al., 2020). Variation also exists in affect (e.g., pleasantness, activation; e.g., Wilson-Mendenhall et al., 2015) and appraisals (e.g., novelty, control; e.g., Kuppens et al., 2003), and variability in all these is magnified by known individual and cultural differences (e.g., Hoemann et al., 2023; Mesquita, 2022). While few would deny the existence of variation in emotion by situation, person, and culture, studies are still rarely designed to look for it (Barrett, 2022). In this paper, we consider what might be discovered if emotion were systematically studied by sampling people deeply ‘in the wild’ using biologically-triggered experience sampling – a multimodal and idiographic approach to ambulatory sensing that links body and mind. We outline the rationale for this approach, propose opportunities for innovation with emerging technologies, and consider the challenges and possibilities this approach brings to the frontier of affective science.

## Biologically-Triggered Experience Sampling

Instances of emotion arise as a complex ensemble of features. Some of these features are biological (e.g., physiological and chemical changes, skeletomotor movements), some are mental (e.g., goals, appraisals, affect), and some are contextual (e.g., environmental conditions, social interactions; Barrett, 2022). These features are in constant interplay with one another. For example, changes in immune function influence thoughts and feelings, which in turn influence interpersonal outcomes (for discussion and relevant references, see Barrett, 2017; Shaffer et al., 2022). Interrelationships such as these emerge as the brain coordinates and regulates the internal bodily systems to meet the predicted metabolic needs required to interface with an ever-changing external world – a process known as allostasis (Sterling, 2012). Allostasis is a brain-wide phenomenon (Barrett & Simmons, 2015; Kaplan & Zimmer, 2020; Kleckner et al., 2017; Sennesh et al., 2022; Sterling & Laughlin, 2015) and all experiences (e.g., perceptions, cognitions, emotions) are conditioned on it (Barrett, 2017; Ganzel et al., 2010). The central role of allostasis in emotion also provides an explanation for the observed variation in the features of these experiences within and across individuals: the activity of various bodily systems is being coordinated in a highly context-sensitive manner, to meet immediate situation-specific needs.

This variation has practical implications for study design, measurement, and analysis in affective science. Situation-dependent relationships between biological and mental features require methods that densely sample signals from multiple modalities (e.g., self-report, physiology, movement). However, most of what is known about emotion is based on instances elicited at a single point in time in lab-based settings via stimuli that do not reflect the complex variations and dynamics of the real world. Studies using experience sampling and other ambulatory sensing methods solve this problem by capturing momentary experiences over time and in daily life. In principle, these methods maximize the diversity of experiences that can be observed (Ibanez, 2022; Wilhelm & Grossman, 2010), and make it possible to characterize context- and/or person-specific patterns in features. Yet most experience sampling studies rely on randomly triggered prompts that are not guaranteed to capture instances of emotion or other allostatically-relevant changes in the body and may miss rarer, affectively intense instances.

Biologically-triggered experience sampling addresses this gap by assessing mental and contextual features at moments when there are notable changes in ongoing biological activity. By initiating sampling based on these shifts in bodily conditions, this approach can more selectively target instances of emotion as they occur throughout daily life. In our own proof-of-concept study (Hoemann et al., 2020), continuous electrocardiography (ECG), impedance cardiography (ICG), and accelerometry were collected and used to trigger prompts whenever an integrated smartphone app detected sustained increases or decreases in the time interval between heartbeats (interbeat interval; IBI) in the absence of major movement or posture change. At each prompt, participants reported on their current experience, including freely-generated emotion labels and rated valence and arousal. Consistent with past work showing pervasive variation in emotion, our unsupervised machine learning analyses revealed patterns of physiological change that varied across individuals and that mapped in a many-to-many relationship with emotion words and affect ratings (Hoemann et al., 2020). Other recent studies have examined triggering prompts based on increases in electrodermal activity (EDA; Van Halem et al., 2020), decreases in heart rate variability (HRV; Schwerdtfeger & Rominger, 2021), and motoric features such as posture and gait (Giurgiu et al., 2020; Kanning et al., 2021).

## Opportunities for Innovation

Relative to existing lab-based and ambulatory approaches, biologically-triggered experience sampling generates longitudinal, within-person data sets that integrate multiple modalities around biological changes of interest. The field has much to gain from this approach, especially as new measures become available and easier to use. In this section, we highlight two opportunities for innovation, where biologically-triggered experience sampling can make a substantive contribution to understanding emotion.

First, increasing the number of modalities sampled will make it possible to model emotion in higher dimensionality, and answer fundamental questions about how biological, mental, and contextual features are related over time. Our initial implementation of biologically-triggered experience sampling, for example, could be augmented by existing and emerging ambulatory measures to track meaningful emotional changes in daily life. Table 1 provides example technologies and the feature(s) monitored by each. These additions can also push forward translational and clinical research. Because the brain continually performs allostasis and all experience relies on this basic process, allostatic dysregulation is coming to be understood as a transdiagnostic vulnerability to mental and physical disorders. Consistent with this hypothesis, growing evidence demonstrates that symptoms of major depressive disorder (e.g., distress, context insensitivity, motor retardation) are associated with persistent problems in energy regulation (Shaffer et al., 2022; see also Tian et al., 2023). Coupling ambulatory sensing technologies that assess immunologic and metabolic function (Table 1) with those assessing peripheral physiological (e.g., cardiovascular) activity can further the longitudinal and situated study of brain-body health.

**Table 1:** Emerging ambulatory sensing technologies

Technology	Features monitored	Exemplar Reference
Electrodermal activity (EDA) sensors integrated into shoes or socks	Long-term EDA	Kappeler-Setz et al., (2013)
Wearable biometric vests	Heart rate (HR) and heart rate variability (HRV), respiration rate and volume, step count and cadence	Haddad et al., (2020)
Multimodal earbud sensors	Photoplethysmography (PPG), accelerometry, core body temperature	Rahman et al., (2022)
Smartphone sensors and applications that capture situated behavior	Mobility, sleep, communication habits	Harari et al., (2017)
Smartphone sensors that assess the external environment	Location, weather, carbon dioxide concentration	Scholz et al., (2017)
Blood pressure (BP) estimated using smartphone optical sensors	On-demand BP	Gordon and Mendes (2021)
Mobile eye tracking	Fixations	Dillen et al., (2020)
Mobile electroencephalography (EEG)	Event-related potentials (ERPs; e.g., P300), frequency band power/asymmetry	Bleichner and Debener (2017)
Continuous glucose monitoring (in people without diabetes)	Real-time glucose levels	Liao and Schembre (2018)
Skin-interfaced wearables that collect eccrine sweat	Momentary cortisol, cytokines	Ghaffari et al., (2021)
Ingestible capsules that assess gastrointestinal function	Gut pressure, pH, temperature	Monti et al., (2021)

Second, innovations in biologically-triggered experience sampling offer a unique opportunity to create and test real-time, person- and context-specific interventions, which can revolutionize the detection and management of stress or even clinical symptomology. Recent studies have combined EDA with skin temperature data to predict stress responses in situ (Kyriakou et al., 2019), used HRV to identify moments when there may be heightened psychological vulnerability (Schwerdtfeger & Rominger, 2021), and tracked self-reported stress in everyday life alongside smartphone-estimated blood pressure (Gordon & Mendes, 2021). These innovations enable targeted sampling of stressful experiences, and can also inform the delivery of just-in-time adaptive interventions (e.g., Nahum-Shani et al., 2018; Schneider et al., 2023). Using biological signals to push content when people are exhibiting increased physiological arousal can supply tools when they may be most effective. Such interventions could ultimately scaffold people’s flexible application of emotion regulatory strategies, by helping them use the right tool for the situation (Blanke et al., 2020; Kalokerinos et al., 2019). If content included information about ongoing biological activity, this may enhance skills such as emotional granularity (i.e., emotion differentiation) by making people aware of features they can use to distinguish different types of emotion experiences in different contexts (Hoemann, Nielson et al., 2021).

## Challenges and Possibilities

Biologically-triggered experience sampling involves practical, methodological and pragmatic challenges. Table 2 outlines considerations for key decision points in study design and data analysis. More generally, a multimodal and idiographic approach to ambulatory sensing requires an interdisciplinary research team, well-organized pipelines for processing, integrating and curating data, and participants who are willing to be intensively sampled and maybe even interested in the results (e.g., Gordon & Mendes, 2021). It also requires augmenting the funding mechanisms that support this research vision, creating different incentive structures within the practice of science, and changing the ways we train the next generation of scientists. The scale of these challenges requires system-level innovation and so requires buy-in from the field writ large.

**Table 2:** Decision points for biologically-triggered experience sampling

Decision point	Example solution from Hoemann et al., (2020)	Considerations
*Study Design*		
How many and which modalities will be sampled?	Electrocardiography (ECG) and impedance cardiography (ICG) were sampled because cardiovascular activity is intrinsically linked to current or anticipated actions that support allostasis (see Obrist et al., 1970). Skeletomotor movement (accelerometry) and posture (inertial measurement units) were also measured to target sampling moments without forward motion or posture change.	The specific modalities needed will depend on the research question. In general, the more modalities sampled, the greater the participant burden – especially if modalities are sampled from separate devices. Multimodal devices (e.g., biometric vests) can help alleviate burden, as can repurposing existing devices (e.g., smartphone) and selecting technologies with novel form factors that enhance wearability (e.g., sock sensors; see Table 1).
Which measure(s) will be used for triggering prompts?	Interbeat interval (IBI) was used because changes in IBI have been linked to changes in subjective experience (Bradley & Lang, 2000) and because IBI is derived from a signal (ECG) that is relatively easy to collect and for which automated processing pipelines are well-established (Nabian et al., 2018).	Changes in end organ function (e.g., IBI) can result from sympathetic activity alone, parasympathetic activity alone, or a combination of the two (Berntson et al., 1994). Studies may want to target measures that reflect activity in only one branch of the autonomic nervous system. Prompts could also be triggered based on conjoint changes in two or more biological measures or between biological and contextual measures.
What values will be used to trigger prompts; how much does the measure need to change and over what period?	A standard starting threshold was used for all participants (i.e., IBI change of ± 167 ms over an 8 s period), and then adjusted at the end of each day for each participant, to ensure everyone received a similar number of prompts per day.	The precision of biological triggers can be increased by setting idiographic thresholds in an automated and adaptive way (Rominger & Schwerdtfeger, 2022; Schwerdtfeger & Rominger, 2021). Studies can also monitor signals using statistical process control tools trained on data from a person-specific baseline or learning phase (Snippe et al., 2023).
*Data Analysis*		
How will missing data be handled?	Only physiological signals around completed experience sampling prompts were sampled and all data from prompts without usable ECG signal were excluded (as this was the basis for triggering prompts and estimating the cardiovascular features modeled). Participants with fewer than 70 prompts devoid of major physiological artifact were also excluded from analysis.	Participants may not respond to prompts, and these non-responses may be systematically related to the sampling design (e.g., if prompts are triggered for possible stress) rather than missing at random (see Wrzus & Neubauer, 2022). Recent work provides ways of addressing non-responsivity in event-contingent experience sampling, for example by including random prompts in study design and modeling (e.g., Ma et al., 2022).
How will baseline differences be handled?	Change scores were calculated for each experience sampling prompt as the difference in cardiovascular activity between the 30 s preceding the IBI change that initiated the prompt and the 30 s following.	Person-specific baselines and thresholds for data exclusion can also be derived from in-lab measures as well as ambulatory measures collected at moments of seated rest (e.g., Hoemann, Khan et al., 2021).
How will interrelationships between measures or modalities be modeled?	Six cardiovascular features were submitted to Bayesian analyses that discovered the number of probabilistic clusters in each person’s data (Bishop, 2006; Blei & Jordan, 2006).	Dynamical systems approaches, such as state-space modeling (Lodewyckx et al., 2011) and recurrence quantification analysis (Coco et al., 2021) can be used to understand non-linear and sometimes weak associations between variables. Time-varying network analysis (Fan et al., 2019; Haslbeck & Waldorp, 2020) can also account for multiple simultaneous dependencies.

Human experience emerges amid dynamically changing signal arrays in the brain, in the body, and from the world. Sampling and modeling this landscape are challenging, but necessary to understand the nature of emotion. While each of the innovations outlined above adds incremental value to the study of emotion in the wild, their real power will be realized from implementing them in concert. A robust, generalizable science of emotion requires sampling individuals across multiple situations in daily life beyond the standardized and restricted experiences evoked in the lab, measuring deeply by simultaneously monitoring mental (e.g., affect, appraisals), biological (e.g., metabolic activity, posture), and contextual features (e.g., ambient temperature, social interaction), and leveraging analytic approaches that capture complex yet reliable patterns of feature variation. Such an approach is required if we are to determine how different features structure the variation in emotion, allowing affective science to reveal generalities rather than presume them.
